# Metabolic syndrome and its associated factors among type 2 diabetic patients in Mizan-Tepi University Teaching Hospital, Southwest Ethiopia Region

**DOI:** 10.3389/fcdhc.2023.1234674

**Published:** 2023-09-13

**Authors:** Abel Shita, Habtamu Teshome, Mulugeta Ayalew, Wudu Yesuf, Dawit Getachew

**Affiliations:** ^1^ Department of Public Health, Mizan Aman College of Health Science, Mizan Aman, Ethiopia; ^2^ Department of Public Health, School of Public Health, Mizan Tepi University College of Health Science, Mizan Aman, Ethiopia

**Keywords:** metabolic syndrome, type 2 diabetes mellitus, Southwest Ethiopia, Mizan Tepi University, prevalence

## Abstract

**Background:**

Patients with diabetes mellitus (DM) are prone to modifiable and non-modifiable complications, which can be grouped under metabolic syndrome (MetS). Evaluating MetS in patients with diabetes is critical for the prevention of cardiovascular disease among patients with DM. In Ethiopia, more specifically in the southwest of Ethiopia, these kinds of information are lacking. Thus, this study estimated the prevalence of metabolic syndrome among type 2 diabetic patients and its associated factors.

**Methods:**

A health facility-based cross-sectional study was done from May 1 to 30, 2021. The data were collected using structured questionnaires, laboratory investigations, and anthropometric measurements. MetS was diagnosed using the modified International Diabetic Federation guidelines (IDF) and the National Cholesterol Education Program Adult Treatment Panel III (NCEP-ATP III) criteria. The data was entered into Epidata and analyzed using SPSS software. Bivariable and multiple variable logistic regression was done to identify the factors associated with MetS. In multiple-variable logistic regression analysis, variables that have a p-value ≤ 0.05 were declared to have statistical significance.

**Result:**

The majority (31.4%) of study participants were within the age group of 41-50 years and the mean ± SD of age is 51.75 ± 11.66, and 54.9% of them were men. In this study, the prevalence of MetS was 31.4% and 41.2% using the IDF and NCEP-ATP III criteria, respectively. Being a woman (AOR = 11.33, 95% CI; 3.73, 34.34; p < 0.001), having a lower level of education (AOR=7.10, 95% CI; 1.88, 26.70; p <0.004), and performing high physical activities (AOR=0.08, 95%CI; 0.01, 0.40; p <0.002) were significantly associated with MetS.

**Conclusion:**

According to this study, the magnitude of Metabolic Syndrome in Mizan-Teppi University Teaching Hospital was 31.4% and 41.2% using IDF and NCEP-ATP III criteria, respectively. Being a woman and having a lower level of education increased the odds of MetS among patients with DM while performing high physical activities decreased the odds of MetS among patients with DM. Therefore, to prevent metabolic syndrome among type 2 DM patients in the study area, it is crucial to focus on women and individuals who have not had access to adequate education. One way to do this is by prioritizing interventions that involve physical activity.

## Introduction

Metabolic syndrome (MetS) according to the International Diabetes Federation is a medical condition characterized by the presence of at least three of five risk factors that are interrelated. These factors include elevated triglyceride levels, low levels of high-density lipoprotein (HDL) cholesterol, high blood pressure (systolic or diastolic), high levels of fasting blood glucose, and central obesity (1). The rise in the prevalence of MetS worldwide increases the risk of CVD and other non-communicable chronic disorders (2).

Diabetes is a chronic medical condition that develops when either the pancreas fails to produce adequate amounts of insulin or the body is unable to use insulin effectively. Diabetes is a significant public health issue and is one of four non-communicable diseases (NCDs) that world leaders have prioritized for action. In recent decades, both the number of diabetes cases and their prevalence have been steadily increasing (3).

The majority of research suggests that metabolic syndrome is most commonly associated with cardiovascular disease as an adverse outcome. However, there is debate about whether type 2 diabetes mellitus is also a significant outcome of metabolic syndrome or one of its components. Several reports have demonstrated a clear link between metabolic syndrome and the onset of diabetes. Additionally, the greater the presence of the MetS components, the higher the mortality rate for CVD (4–6).

Nearly 70–80% of the population with DM was diagnosed with metabolic syndrome (7). Worldwide, 20–25% of adults are expected to have MetS. Individuals with MetS are twice as likely to die and three times as likely to have a heart attack or stroke when compared to people without MetS (8). This indicates the co-existence effect of type 2 DM and MetS on cardiovascular risks (9).

There have been studies conducted in Ethiopia that indicate the presence of certain components of MetS among individuals with type 2 diabetes mellitus. Specifically, these studies report hypertension in 46.5% of patients, obesity in 23.4%, and dyslipidemia in 63.5% (10). Another study found that 19.6% of the population surveyed in Ethiopia had hypertension (11). Despite these findings, there has been limited research conducted to evaluate the prevalence and potential risk factors for the development of MetS among type 2 diabetes mellitus patients. Among these, a few studies were conducted to assess the prevalence of MetS in relation to gender in diabetic patients (12).

Strictly diagnosing MetS and its components in DM patients is vital to promote patients’ health care and minimize CVD-related morbidity and mortality. Accordingly, studies are available regarding the prevalence and rising trends of type 2 DM in Sub-Saharan Africa. However, studies on the prevalence of MetS and its associated factors among type 2 DM patients are still inadequate in developing countries including Ethiopia.

Thus, this study assessed the prevalence of MetS and the extent of its components among type 2 diabetes mellitus patients receiving care at Mizan Tepi University Teaching Hospital (MTUTH) in Southwest Ethiopia people’s regional state. Health professionals and program managers can use the information to create and implement effective interventions for MetS prevention and control among diabetic patients. In addition, this research can serve as a baseline for future studies in this area.

## Methods and materials

### Study area and period

The study was carried out at Mizan Tepi University Teaching Hospital from May 1 to 30, 2021. The university hospital was founded in the recently established Southwest People’s Regional State of Ethiopia (SWEPR), which is the 11th region of the nation. The region has six administrative zones: Kaffa, Bench-Sheko, Sheka, West-Omo, Dawro, and Konta. The area as a whole is inhabited by roughly 3.5 million people and has 134 health centers, 836 health posts, 2 general hospitals, and 10 primary-level hospitals, but only 1 teaching hospital. Moreover, this teaching hospital also functions as a referral center for the nearby Gambela region, refugee camps, and even neighboring countries like South Sudan.

### Study design

A health facility-based cross-sectional study design was done to assess the prevalence of MetS and the extent of its components among type 2 diabetes mellitus patients receiving care at Mizan Tepi University Teaching Hospital.

### Population

The source population were all patients with type 2 diabetes mellitus who were on follow-up at the clinic in Mizan Tepi University Teaching Hospital and the study population were all patients with type 2 diabetes mellitus attending follow-up clinics in Mizan Tepi University Teaching Hospital during the study period. Patients with a history of other chronic diseases, those receiving lipid-lowering treatment, pregnant women, and lactating mothers were excluded from the study.

### Sample size and sampling procedure

The sample size was calculated based on a single population proportion formula taking a 95% confidence interval (CI) and 66.7% prevalence of metabolic syndrome among type 2 DM patients from a study in North West Ethiopia (13). With this assumption, the final calculated sample size was 341. The total number of type 2 DM patients attending follow-up at MTUTH during the study period was 239 which was less than the calculated sample size. Therefore, type 2 DM patients attending the follow-up clinic within the study period (204) and who fulfilled inclusion criteria were consecutively recruited into the study.

### Variables

The dependent variable in this study is MetS. The independent variables comprise four categories, which are socio-demographic (age, gender, education, income, and employment status), behavioral (substance use, physical activity, and dietary habit), physical measurements (blood pressure, height, weight, waist, and hip circumference), and biochemical measurements (triglyceride level, HDL level, and blood glucose level).

The Global Physical Activity Questionnaire (GPAQ), developed by the World Health Organization (WHO), was used to measure physical activity in this study. Metabolic equivalent tasks (MET) in minutes (MET-min) per week were calculated from the physical activity domains, and these were categorized as low physical activity (0 to 600 MET-min per week), moderate physical activity (601–3000 MET-min per week), and high physical activity (3000 MET-min per week) (14).

### Data collection tools and procedures

A structured questionnaire adapted from the WHO STEPS surveillance manual for non-communicable disease risk factors was used. The anthropometric measurements and interviews were performed by four trained BSc Nurses, and the questionnaire was pretested on 10% of the sample size at another Hospital. The laboratory investigations were carried out by two trained laboratory technologists, while the data collection was supervised by two trained MPH holders. COVID-19 prevention protocols were adhered to during the data collection, with both the participants and data collectors wearing face masks and using sanitizer as needed.

### Measurements

#### Anthropometric measurements

All anthropometric measurements were performed according to the WHO stepwise approach to NCDs risk factor surveillance (15). Accordingly, blood pressure was measured after the participants rested with legs uncrossed for 15 minutes, using a standard adult arm cuff electronic sphygmomanometer. The left arm was placed on the table and the cuff was positioned 1-2cm above the elbow point. Three readings were taken and the mean of the second and third readings was calculated for data analysis.

A stadiometer was used to measure height, with participants removing footwear and headgear. They stood on the board with feet together and eyes looking straight ahead, while height was recorded in centimeters to the nearest 0.1 cm with accuracy ensured by placement against a firm wall surface.

Weight was measured with a digital scale placed on a firm, flat surface. Participants removed footwear, socks, and heavy belts, and emptied their pockets of mobiles, wallets, and coins. Participants stood facing forward with arms at their sides for weight measurement in kilograms to the nearest 0.1 kg.

A constant tension tape was used to measure waist circumference. Participants removed any thick clothing, and the measurement was taken at the midpoint between the lower margin of the last palpable rib and the top of the iliac crest (hip bone) with arms relaxed and at the end of a normal expiration.

Hip circumference was measured using a constant tension tape while the participant’s arms are relaxed at the sides at the maximum circumference over the buttocks. Thick or bulky items of clothing were removed.

BMI (kg/m2) was calculated by dividing weight (in kg) by height squared (in m2) (14). This was used to classify participants into underweight (BMI < 18.5), normal weight (BMI 18.5-24.9), overweight (BMI 25-29.9), and obese (BMI ≥ 30) categories.

#### Biochemical measurements

Blood samples were collected from participants in the early morning before they took any meal and after 12 hours of fasting. Fasting blood samples of 5 ml were collected in plain test tubes and serum was extracted. The extracted serum was analyzed for glucose and lipid profile levels using Bio Systems A25. Triglycerides, HDL-c, LDL-c, and total cholesterol were determined using specific enzymatic methods, and glucose was determined using the glucose oxidase method.

### Definition of metabolic syndrome

Using the IDF criteria, patients were diagnosed with MetS if they have abdominal obesity (waist circumference of ≥ 94cm for men and ≥ 80cm for women) and at least two of the following components: elevated triglyceride levels (≥ 150mg/dL), lower levels of high-density lipoprotein cholesterol (< 40 mg/dL in men and < 50 mg/dL in women), high blood pressure (systolic blood pressure ≥ 130 mmHg or diastolic blood pressure ≥ 85 mmHg), or a previous diagnosis of hypertension that is being treated (16).

Based on the National Cholesterol Education Program Adult Treatment Panel III (NCEP-ATP III) criteria, patients were classified as having MetS if they had three or more of the following four risk factors: abdominal obesity (waist circumference >102 cm in men and >88 cm in women), TG (≥150 mg/dl), reduced HDL-c ((<40 mg/dl in men and <50 mg/dl in women), and high arterial BP (≥130/85 mmHg) (17)

### Data quality assurance

To ensure data quality, the data collectors were trained, and the questionnaire was pretested and modified based on the findings prior to actual data collection. The collection process was closely supervised, and the questionnaire was regularly reviewed and checked for logical consistency and completeness.

### Data processing and analysis

The data underwent coding, cleaning, and entry into EpiData version 4.4.3.1 before being analyzed with SPSS V.25. Descriptive statistics were presented for numeric variables using mean and standard deviation, while categorical variables were presented as percentages. In the logistic regression model, variables that had an association with the dependent variable in bivariable analysis at a p-value ≤ 0.2 were considered candidates for the multiple logistic regression model. Finally, variables with a p-value ≤ 0.05 were considered as having a statistically significant association with MetS.

## Result

### Socioeconomic status of participants

The study involved 204 type 2 patients with diabetes mellitus. Out of this, 112 (54.9%) of the study participants were men, 92 (45%) of them were orthodox religious followers, and a majority [176 (86.3)] were married. Nearly one-third [75 (35.3%)] of the participants had no formal education followed by 52 (25.5%) who could read and write. The majority [168 (82.4%)] of the participants were urban residents and 68 (33.3%) were merchants. Sixty-four (31.4%) of them were within the age group of between 41 and 50 years old (See **Table 1**).

**Table 1:** Socio-economic characteristics of type 2 DM patients attending the MTUTH follow-up clinic, 2021.

Variables	Categories		Number	Percent (%)
Sex	Men		112	54.9
	Women		92	45.1
Age in year	30-40		36	17.6
	41-50		64	31.4
	51-60		56	27.5
	61-90		48	23.5
	Mean ± (SD) = 51.75 ± (11.66)			
Level of education	No formal education		72	35.3
	Read and write		52	25.5
	Primary/Secondary School		40	19.6
	Diploma and above		40	19.6
Residence	Rural		36	17.6
	Urban		168	82.4
Marital status	Married		176	86.3
	Widowed		20	9.8
	Single/separated		8	3.9
Occupation	Government employee		52	25.5
	Merchant		68	33.3
	Housemaid		56	27.5
	Farmer		20	9.8
	Job seeker/unemployed		8	3.9
Religion	Orthodox		92	45.1
	Muslim		48	23.5
	Protestant		64	31.4

### Behavioral measurements of study participants

Out of 204 patients with type 2 DM, 196 (96.1%) reported having never smoked cigarettes, 104 (51%) have never drunk alcohol, 48 (23.5%) had consumed alcoholic drinks within the past 12 months, and 48 (23.5%) had consumed alcohol within the past 30 days. Additionally, 160 (78.4%) patients reported recent coffee consumption, while 28 (13.7%) reported ever having chewed khat within the past 12 months. Forty-four (21.6%) of the participants perform high physical activities in a typical week, 124 (60.8%) perform moderate physical activities in a typical week, and 156 (76.5%) of them walk for at least 10 minutes per day in a typical week (see **Table 2**).

**Table 2:** Behavioral characteristics of type 2 DM patients attending the MTUTH follow-up clinic, 2021.

Variables	Categories	Number	Percent
Ever smoked cigarette	Yes	8	3.9
	No	196	96.1
Ever consumed an alcoholic drink	Yes	100	49.0
	No	104	51.0
Consumed alcohol within the past 12 months?	Yes	48	23.5
	No	156	76.5
Frequency of alcoholic drinks within the past 12 months	Daily	0	0.0
	5-6 days per week	16	25.0
	3-4 days per week	16	25.0
	1-2 days per week	16	25.0
	1-3 days per month	8	12.5
	Less than once a month	4	6.3
	I have not	4	6.3
Consumed at least one standard alcohol in the past 30 days	Yes	51	25.0
	No	153	75.1
Drink coffee recently?	Yes	160	78.4
	No	44	21.6
Ever chewed khat in the past 12 months?	Yes	28	13.7
	No	176	86.3
Performs high physical activities	Yes	44	21.6
	No	160	78.4
Performs moderate physical activities	Yes	124	60.8
	No	80	39.2
Days of walking for at least 10 minutes in a typical week	5	24	11.8
	6	24	11.8
	7	156	76.5

### Clinical, physical, and biomedical profiles

Out of all the participants in the study, 52 (25%) of them have a family history of diabetes mellitus. Of these individuals, 108 (52.9%) are currently taking anti-hypertensive drugs, and 100 (49%) were diagnosed with type 2 DM within the last 5 years. While 96 participants (47.1%) have a normal body mass index, the others are either overweight or obese.

Approximately Seventy-two (35.3%) and 40 (19.6%) of the participants have central obesity according to the IDF criteria and NCEP ATP III criteria, respectively. Additionally, nearly one-fourth of them (25.5%) have triglyceride levels at or above 150, 52 (25.5%) have low levels of high-density lipoprotein (HDL), and 152 (74.5%) have a blood pressure of SBP≥130 and or DBP≥85 (see **Table 3**).

**Table 3:** Clinical, physical, and biomedical characteristics of type 2 DM patients attending the MTUTH follow-up clinic, 2021.

Variables	Categories	Number	Percent
Family history of DM	Yes	52	25.5
	No	152	74.5
Currently taking an anti-hypertensive drug	Yes	108	52.9
	No	96	47.1
Duration since the diagnosis of DM	<1 year	12	5.9
	>1-5 years	100	49.0
	5-6 years	36	17.6
	6-10 years	44	21.6
	>10 years	12	5.9
Body mass index	Underweight	0	0.0
	Normal	96	47.1
	Overweight	72	35.3
	Obese	36	17.6
Central obesity (IDF)	Yes	72	35.3
	No	132	64.7
Central obesity by NCEP ATP III criteria	No	164	80.4
	Yes	40	19.6
Triglyceride	TG<150	152	74.5
	TG>=150	52	25.5
Low level of HDL	No	152	74.5
	Yes	52	25.5
Blood pressure: (SBP≥130 DBP≥85)	No	52	25.5
	Yes	152	74.5

### Prevalence of MS

**Figure 1:**
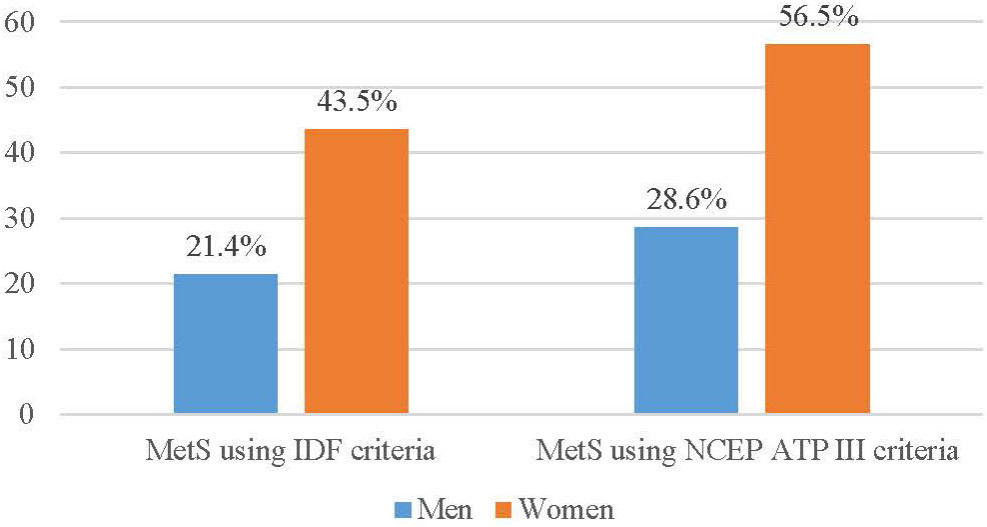
Prevalence of metabolic syndrome among type 2 DM patients attending follow-up clinic at MTUTH in May 2021.

The prevalence of MetS among those with type 2 diabetes using the IDF criteria was 64 (31.4%) and it was 84 (41.2%) using the NCEP-ATP III criteria. Additionally, when looking at the prevalence based on sex, using the IDF criteria, 24 men (21.4%) and 40 women (28.6%) have MetS while using the NCEP-ATP III criteria, 32 men (28.6%) and 52 women (56.5%) have MetS (see **Figure 1**).

### Factors associated with MS among type 2 diabetic patients

In this study, the metabolic syndrome level which is calculated by IDF criteria was used to dichotomize for bivariate and multivariable analysis. In a bivariate analysis, the sex of the participant, level of education, alcohol consumption, performance of high physical activity, and performance of moderate physical activity were associated with MetS at a p-value <0.2. In multiple logistic regression, being a woman, having a lower level of education, and performing high physical activities were significantly associated with MetS at a p-value < 0.05 (see **Table 4**).

**Table 4:** Bivariable and Multiple variable logistic regression analysis of the metabolic syndrome among type 2 DM patients attending the MTUTH follow-up clinic, 2021.

Variables category		Yes N (%)	No N (%)	COR (95% CI)	AOR(95% CI)
Sex	Men	24 (21.4)	88 (78.6)	1	1
	Women	40 (43.5)	52 (56.5)	2.82 (1.53, 5.19)*	11.33 (3.73, 34.34)**
Occupation	Government employee	12 (23.1)	40 (76.9)	1	
	Merchant	28 (41.2)	40 (58.8)	2.33 (1.04, 5.22)	
	Housemaid	24 (42.9)	32 (57.1)	2.50 (1.08, 5.75)	
Residence	Rural	8 (22.2)	28 (77.8)	1	1
	Urban	56 (33.3)	112 (66.7)	1.75 (0.74, 4.08)*	0.36 (0.06, 1.97)
Age in years	30-40	12 (33.3)	24 (66.7)	1	
	41-60	36 (30.0)	84 (70.0)	0.85 (0.38, 1.89)	
	61-90	16 (33.3)	32 (66.7)	1.00 (0.40, 2.50)	
Level of education	No formal education	16 (22.2)	56 (77.8)	0.66 (0.27, 1.60)	0.37 (0.10, 1.28)
	Read and write	28 (53.8)	24 (46.2)	2.72 (1.14, 6.48)*	7.10(1.88,26.70)**
	Primary/Secondary	8 (20.0)	32 (80.0)	0.58 (0.20, 1.63)*	1.10 (0.25, 4.87)
	Diploma and above	12 (30.0)	28 (70.0)	1	1
Ever been alcoholic	Yes	24 (24.0)	76 (76.0)	0.50 (0.27, 0.92)*	0.73 (0.26, 2.02)
	No	40 (38.5)	64 (61.5)	1	1
Alcohol in 12 months	Yes	12 (25.0)	36 (75.0)	1.11 (0.44, 2.78)	
	No	12 (23.1)	40 (76.9)	1	
Alcohol in 30 days	Yes	12 (25.0)	36 (75.0)	0.66 (0.17, 2.61)	
	No	4 (33.3)	8 (66.7)	1	
Drank coffee recently	Yes	52 (32.5)	108 (67.5)	1.28 (0.61, 2.69)	
	No	12 (27.3)	32 (72.7)	1	
Ever chewed khat	Yes	8 (28.6)	20 (71.4)	0.85 (0.35, 2.06)	
	No	56 (31.8)	120 (68.2)	1	
High physical activity	Yes	4 (9.1)	40 (90.9)	0.16 (0.05, 0.48)*	0.08 (0.01, 0.40)**
	No	60 (37.5)	100 (62.5)	1	1
Moderate physical activity	Yes	32 (25.8)	92 (74.2)	0.52 (0.28, 0.95)*	0.63 (0.27, 1.47)
	No	32 (40.0)	48 (60.0)	1	1
Family history of DM	Yes	16 (30.8)	36 (69.2)	0.96 (0.48, 1.90)	
	No	48 (31.6)	104 (68.4)	1	
Duration since the diagnosis of DM	<1 year	4 (33.3)	8 (66.7)	1.00 (0.18, 5.46)	0.80 (0.09, 6.64)
	>1-5 years	32 (32.0)	68 (68.0)	0.94 (0.26, 3.35)	0.58 (0.13, 2.46)
	5-6 years	4 (11.1)	32 (88.9)	0.25 (0.05, 1.22)*	0.38 (0.05, 2.63)
	6-10 years	20 (45.5)	24 (54.5)	1.66 (0.43, 6.35)	2.83 (0.58, 13.73)
	>10 years	4 (33.3)	8 (66.7)	1	1

According to this study, being a woman increased the odds of MetS among type 2 DM patients by 11 times compared to men with type 2 DM (AOR = 11.33, 95% CI; 3.73, 34.34; p <0.001). Also, the odds of developing metabolic syndrome are seven times higher among type 2 DM patients that can only read and write compared to those patients having diplomas and above (AOR=7.10, 95%CI; 1.88, 26.70; p <0.004). In comparison to type 2 DM patients who do not engage in high physical activity in a typical week, those who do moderate physical activity have a significant 92% decrease in the likelihood of developing MetS (AOR=0.08, 95%CI; 0.01, 0.40; p <0.002).

## Discussion

According to this study, based on the criteria set by the International Diabetes Federation (IDF), the prevalence of MetS in type 2 diabetic patients was 31.4% (25.3%-38.0%). This figure is relatively lower than other studies conducted in various countries. Ethiopia, Pakistan, Italy, Libya, Bangladesh, and Sri Lanka have reported rates ranging from 61% to 80.8% (18–22). The difference could be changes in lifestyle and cultural factors. In this study, being a woman increased the odds of MetS among type 2 DM patients by 11 times compared to men with type 2 DM. The results of this study are consistent with those of previous studies conducted at Dessie Referral Hospital, as well as Mekelle Ayder Hospital and Gonder University Hospitals (13, 23, 24). This higher rate of MetS among women may be due to the sedentary lifestyle that most women lead and genetic or age variation (7, 25). Supporting this evidence, our study found that the prevalence of MetS using IDF criteria is 62.5% among women compared to 37.5% among men. Additionally, 76.8% of women had central obesity compared to 23.3% of men, and 69.2% of women had reduced HDL compared to 30.8% of men.

Also, the odds of developing metabolic syndrome are seven times higher among study participants that can only read and write compared to those patients who have an educational status of diploma and above. The reason could be that individuals with lower educational status are likely to have limited access to healthcare and health education and have an unhealthy lifestyle due to a lack of knowledge or awareness (26).

Moreover, the odds of MetS in study participants who engage in high physical activity in a typical week decreased by 92% compared to those who do not engage in high physical activity. This is in line with study results in Ethiopia (22, 23, 27).

## Strengths and limitations of the study

The limitations of this study are its cross-sectional nature, which makes it difficult to assess the temporal relationship between variables. Additionally, the study was not conducted in multiple centers. However, as it was conducted at the only referral hospital in the region, it could be considered representative. On the other hand, the use of multiple criteria to measure metabolic syndrome is the strength of this study.

## Conclusion and recommendation

This study has found a high prevalence of metabolic syndrome among type 2 diabetic patients attending follow-up clinics at MTUTH. Being a woman, having a decreased level of education, and not performing high physical activity were all factors associated with MetS. Prioritizing female patients or those with lower levels of education in interventions and physical activity promotion programs could help prevent metabolic syndrome among type 2 DM patients.

## Data availability statement

The original contributions presented in the study are included in the article/supplementary material. Further inquiries can be directed to the corresponding author.

## Ethics statement

The Mizan Aman College of Health Science Research Ethics Review Committee (RERC) gave its approval for this investigation (RERC number: 005/2021) and a support letter was given to MTUTH. After outlining the goals, purpose, and potential risks and benefits of the study, all participants were asked to sign a written consent form before the study could begin. Additionally, throughout the whole study, anonymity, confidentiality, and the right to withdraw from the study at any moment were all upheld. 

## Author contributions

AS and HT wrote the proposal, analyzed the data, and drafted the paper. MA and WY approved the proposal and analysis with some revisions. DG participated in the data analysis and revised subsequent drafts of the paper. All authors contributed to the article and approved the submitted version.
